# Experiences of family caregivers in dealing with cases of advanced breast cancer: a qualitative study of the sociocultural context in Punjab, Pakistan

**DOI:** 10.1186/s12889-024-18404-1

**Published:** 2024-04-12

**Authors:** Sadia Jabeen, Rubeena Zakar, Muhammad Zakria Zakar, Florian Fischer

**Affiliations:** 1 Department of Sociology, Virtual University of Pakistan, Lahore, Pakistan; 2 Institute of Social and Cultural Studies, University of the Punjab, Lahore, Pakistan; 3 University of Poonch, Rawalakot, Azad Jammu and Kashmir Pakistan; 4 Institute of Public Health, Charité– Universitätsmedizin Berlin Institute of Public Health, Charitéplatz 1, 10117 Berlin, Germany

**Keywords:** Breast cancer, Family caregivers, Care, Coping strategies, Sociocultural factors

## Abstract

**Background:**

Patients with advanced breast cancer require consistent help and support from family caregivers. These caregivers often endure financial burdens and psychological stress, with their experiences significantly influenced by sociocultural factors. This study aims to explore the experiences of family caregivers of advanced breast cancer patients in Punjab province, Pakistan.

**Methods:**

Data was collected through in-depth interviews with fifteen family caregivers of advanced breast cancer patients in three major cities of Punjab, Pakistan. Caregivers, who had been in close contact with the patient for the last two years, were purposively sampled from five major hospitals. The data was analyzed using thematic analysis.

**Results:**

The study revealed that the experiences of family caregivers are deeply rooted in the sociocultural context. Key themes identified include social responsibility and cultural reciprocity norms; limited awareness and mobility options for caregivers; financial responsibility and strain; impacts of beauty myths and shyness on caregiving attitudes and decisions; the stressful and emotional nature of caregiving; treatment perspectives influenced by social groups; challenges in consulting male physicians and associated stigma; the role of religious beliefs in caregiving; and stress management, with religion often being a coping mechanism. These factors can contribute to delayed treatment decisions for patients.

**Conclusions:**

Family caregivers are crucial in facilitating timely treatment decisions for advanced breast cancer patients in the Pakistani context. To minimize treatment delays and alleviate caregiver stress, addressing sociocultural barriers in care-seeking is essential. A tailored approach, considering sociocultural and religious factors, is imperative for the management and early diagnosis of breast cancer, necessitating appropriate policymaking and implementation.

## Background

Breast cancer is the leading cause of mortality and the most common malignancy in Pakistan, a country with one of the highest prevalence of the disease in Asia. This condition predominantly affects women, especially those below the age of 60. With an annual age-specific incidence rate of 50.1 per 100,000 individuals, Pakistan’s rate is notably higher than that of other regional countries [1]. It is estimated that one in nine Pakistani women may develop breast cancer in their lifetime, and the country records the highest fatality rates attributed to breast cancer in Asia [2].

The prevalence of breast cancer is highest among all malignancies in Pakistan. Contributing to the high mortality rate are factors such as limited awareness, insufficient early-stage screening resources, and various cultural, gender-specific, and socio-economic influences that impede women’s autonomy in seeking medical care [3]. The social dynamics in Pakistan, encompassing family, friends, and relatives, exert significant influence through financial support, persuasion, and emotional bonds [4]. These dynamics often deter women from seeking necessary healthcare due to marital and familial pressures. As a result, many Pakistani women diagnosed with breast cancer are compelled to conform to family and societal expectations, with decision-making often deferred to caregivers who also possess financial authority [5, 6]. However, these caregivers face their own set of sociocultural and financial challenges.

### Family caregiving in sociocultural context

Cancer has a lifetime effect not only on patients, but also on other family members, particularly on family caregivers who also pass through the different phases along with patients of advanced breast cancer [7, 8]. Previous research has underscored the multifaceted nature of caregiving, encompassing both responsibilities and impacts on daily life [9, 10]. Cancer’s impact extends beyond the patient, significantly affecting family members, especially caregivers. Previous research has underscored the multifaceted nature of caregiving, encompassing both responsibilities and impacts on daily life. In this realm, sociocultural factors play a pivotal role in health-related behaviors, such as care-seeking. The decision-making process in seeking medical consultation is a complex, multi-level phenomenon, influenced by individual traits, family dynamics, and sociocultural values [11, 12]. How women interpret, perceive, and share their health concerns, and how they pursue healthcare consultation are interdependent factors.

Cancer diagnosis impacts not only the patient but also significantly affects family caregivers, who bear the responsibility of caring for their loved ones. The growing trend of outpatient treatment has amplified the role of family and friends in providing not only practical support but also moral and psychological support to patients [13]. Caregivers not only support the patient’s routine care and medical treatment but often must balance their own needs against the demands of caregiving [14, 15]. This balancing act can lead to distress and anxiety, affecting both the caregiving process and the caregiver’s own quality of life. Family caregivers, therefore, are at a higher risk of negative psychological and emotional consequences as the cancer progresses [16, 17].

Despite playing a crucial role in patient care, many caregivers lack adequate information, training, and awareness for handling cancer patients effectively. They often navigate complex healthcare systems with limited support. Unfortunately, intervention programs for caregiver support are scarce, even though there is a clear need for both psychological and social assistance [13].

Family caregivers are increasingly vulnerable to adverse consequences as the cancer progresses to more advanced stages [18]. Existing research highlights how the responsibilities of caregiving can detrimentally affect caregivers, disrupting their daily routines and leading to a cascade of challenges [19]. These include difficulties in managing day-to-day tasks, impediments to professional growth, a decrease in communication with friends and other family members, mental exhaustion, and the overwhelming burden of care. Such factors, particularly the intense focus on the patient’s health, can significantly impair the psychological and emotional well-being of caregivers [18, 19].

Despite playing a crucial role in patient care, many caregivers lack adequate information, training, and awareness for handling cancer patients effectively [18, 19]. They often navigate complex healthcare systems with limited support. Unfortunately, intervention programs for caregiver support are scarce, even though there is a clear need for both psychological and social assistance [20].

### The sociocultural framework of family caregiving

Historically, research on family caregiving has predominantly focused on individual experiences, overlooking the broader sociocultural context. This study aims to fill this gap by examining the sociocultural framework that contextualizes an individual’s position within social, cultural, and dialogical environments [21]. This model facilitates understanding of the caregiving experience at multiple levels, emphasizing motivation, the interdependence of individual, interpersonal, social, and institutional factors [22].

In this framework, the individual caregiver is seen as a central agent, whose actions are contextual within various social relations and settings. This approach recognizes that caregivers’ experiences are shaped by diverse social situations they encounter [9]. This study operates under the assumption that cancer treatment involves not just the patient and healthcare providers, but also the experiences of family members and caregivers. Therefore, exploring the experiences of family caregivers of advanced breast cancer patients within this sociocultural context is crucial to understanding and improving the overall treatment and support process.

## Methods

We conducted a qualitative study adopting the interpretivist philosophical stance. Based on the epistemological stance of social constructionism and ontological stance of interpretivism, phenomenological approach explores the sociocultural context influencing family caregiving behaviors. Social constructionism posits that truth and meaning arise from our engagement with the world, asserting that all knowledge and meaningful reality are contingent upon human practices and interactions within a social context [23]. For that reason, we tried to capture the reality of caregiving experiences within the sociocultural context and the way these experiences are built in this frame.

### Study site

This study was conducted in Punjab province, Pakistan, the second largest and most densely populated province in the country. Given the absence of a comprehensive cancer registry, estimating the exact number of cancer cases is challenging. However, annual cancer registry reports indicate Punjab as having the highest cancer prevalence in Pakistan [24]. In Punjab province, there are nine major divisions. Among these, only three divisions — Lahore, Faisalabad, and Multan — have cancer wards in public sector hospitals. Therefore, our data collection was conducted across these three divisions.

### Data collection

In-depth interviews targeted family caregivers of advanced-stage breast cancer patients who played significant roles in medical decision-making and remained involved throughout the treatment process. The definition of ‘family Caregiver’, in this context, encompasses any relative, partner, friend, or neighbor with a significant personal relationship who provides a wide range of assistance to a person with cancer. These individuals may be primary or secondary caregivers and live with, or separately from, the person receiving care [25]. In Pakistan, these caregivers are considered integral to the functioning of healthcare system [26]. The responsibilities of caregivers can vary depending on several factors. These include the specific type and stage of cancer, its location, the accessibility and quality of healthcare services, as well as the personal and socio-cultural characteristics of both the patient and caregiver, and the nature of their relationship. These responsibilities encompass managing patient care phases, interacting with healthcare professionals, and facilitating secondary care [27]. An interview guide was developed based on deductive codes from prior literature reviews and discussions with caregivers [28]. It included questions about interpersonal relations, sociocultural and community context, and knowledge of caregivers about the disease and the challenges the face in seeking and accessing treatment including associated difficulties.

The guide explored the treatment process, identifying social factors that impede access to treatment and care in nearby health services. It also examined cultural factors that hinder women from seeking and obtaining necessary treatment and care, as well as the influence of women’s status in the community on their decision-making regarding treatment. Additional areas of inquiry included challenges faced while consulting with public or private sector health facilities, issues encountered from friends and the community when taking patients for treatment, emotional and psychological trauma experienced by caregivers, difficulties in hospital management, and interactions with doctors and medical procedures. The guide also sought caregivers’ suggestions on improving health conditions and steps that could be taken to reduce breast cancer cases in Pakistan.

Interviews were conducted by the first author until the saturation point was reached. We obtained lists of stage IV breast cancer patients from oncology wards in five public sector hospitals across the selected divisions. Caregivers who had been in close contact with the patient for a minimum of two years were approached for participation in the study. This included caregivers of both inpatients (those who were admitted to the hospital) and outpatients (those who visited the hospital for chemotherapy or medication and were discharged on the same day). Consent for participation was sought from these caregivers. The inclusion criteria encompassed attendants of patients diagnosed with stage IV breast cancer, who had accompanied the patient for at least two years, were major decision-makers in treatment choices, and bore the financial expenses of the patient. The exclusion criteria were set accordingly.

We obtained lists of stage IV breast cancer patients from oncology wards in five public sector hospitals across the selected divisions.

The timing and location of the interviews were determined through mutual agreement with the participants. Each interview was conducted in a private room to ensure confidentiality and comfort. In each of the five hospitals, three in-depth interviews were carried out, leading to a total of 15 interviews. Each interview spanned approximately 60 to 75 min. Data saturation was achieved after the thirteenth interview. However, to ensure comprehensive coverage and no vital information was overlooked, two additional interviews were conducted. This approach also guaranteed consistency and equal representation from all participating hospitals.

### Data analysis

Interviews were audio-recorded, transcribed, and translated from Punjabi and Urdu into English. Thematic analysis was employed, involving initial coding to identify specific data features relevant to the study question. The data analysis process for this study was meticulously executed to ensure the thorough exploration and accurate representation of the collected data. The researcher diligently listened to the recorded interviews multiple times and meticulously read and re-read the transcribed data to confirm the accuracy of transcription. This initial phase was crucial for establishing a reliable foundation for further analysis.

Following this, the researcher engaged in initial coding, identifying specific features of the data that were directly relevant to the study research questions. The next phase involved pattern recognition, where similar codes were grouped together. This methodical approach facilitated the consolidation of all initial codes that were pertinent to the research objectives, thereby forming distinct themes. The development of thematic maps was a key part of this process, aiding in the generation of themes.

In the course of the analysis, inductive themes naturally emerged from the data, providing insights that were not preconceived. Each theme was then clearly defined and thoroughly explained, ensuring that the analysis was both comprehensive and comprehensible.

The narratives and stories within these themes were considered in relation to the overall context of the research, adding depth and dimension to the analysis. In the final stage of the process, specific examples from the transcripts were carefully selected and elaborated upon. These examples served to clarify and illustrate the themes, making them more accessible and understandable [29, 30].

Throughout the analysis, techniques of constant comparison and analytical induction were employed. This approach allowed for the emergence of sub-themes and categories during the processes of note-taking, transcription, and interpretation, ensuring a rich and nuanced understanding of the data.

In qualitative research, it is very important to express the positionality of the researcher. The researchers’ positionality was critically reflected upon, acknowledging that one’s social position influences perception [31]. The female gender of the researcher affected engagement and communication with participants, leading to an emotional investment in the study. Additionally, having a sound theoretical and social background, the researcher made authentic representations of the stance of participants. Despite sharing a similar cultural background with participants, the diversity of views was respected and authentically represented.

To ensure trustworthiness, we followed Guba and Lincoln’s Four Dimension criteria: credibility, dependability, confirmability, and transferability [32]. Credibility was established through audio recordings of interviews, transcription accuracy of the information, and deep probing during interviews. In order to validate the subjective impression of each interview, the researcher continued getting information until the saturation point. Directly after the interview, nonverbal gestures and reflections were noted against each study participant. Dependability was ensured by repeated listening and detailed information validation. Confirmability was enhanced by discussing findings with a supervisor, and transferability was addressed by providing detailed descriptions of the research process.

### Ethical considerations

This study is a part of the doctoral research of the first author. The study protocol has been reviewed and approved by the Advanced Study and Research Board of the University of the Punjab (No. D/8555/Acad). Researchers took care of ethical consideration while collecting the data considering the matter as personal. During the in-depth interviews, aspects of confidentiality, anonymity, and respect for privacy of respondents were meticulously observed. A written informed consent was obtained from study participants before starting the interviews with the option to terminate the interview at any stage.

## Results

A total of 15 in-depth interviews were conducted with caregivers of advanced breast cancer patients. The age range of respondents spanned from 20 to 60 years, comprising five males and ten females. A majority of these respondents resided in rural areas and reported income between 16,000 and 30,000 Pakistani rupees. Educational level of respondents varied from no formal schooling to graduation, with most having a low level of education (Table 1).

**Table 1:** Sociodemographic characteristics of study participants (*n* = 15)

Variable	n	%
**Age (in years)**		
≤ 25	5	33.3
26–40	8	53.3
≥ 41	2	13.4
**Gender**		
Male	5	33.3
Female	10	66.7
**Relation with patient**		
Son	2	13.3
Brother	3	20.0
Daughter	3	20.0
Mother	2	13.3
Sister	5	33.4
**Types of residence**		
Rural	10	66.7
Urban	5	33.3
**Monthly income of family (in Pakistani rupees)**		
< 16,000	3	20.0
16,000–30,000	8	53.3
> 30,000	4	26.7
**Education**		
Illiterate	2	13.3
1–5 years of schooling grade	3	20.0
6–12 years of schooling	5	33.3
13–14 years of education	3	20.0
Above 14 years of education	2	13.4

We identified several major themes through these interviews, as described in Table 2. These include social responsibility and cultural reciprocity norms; lack of awareness and limited mobility options for caregivers; financial responsibility and strain experienced by caregivers, myths of beauty and shyness influencing caregiving attitudes and decisions; the stressful and emotional experience of caregivers; the impact of treatment perspectives shaped by surrounding social groups, challenges in consulting with male physicians and the associated stigma; and the role of religious beliefs in caring for advanced breast cancer patients and in managing stress.

**Table 2:** List of themes and major codes

Themes	Major codes
Social responsibility and cultural reciprocity norms	▪ Respondents highlighted that caring for advanced breast cancer patients is mainly resulted from family and socio-cultural responsibility. ▪ Respondents also perceive caring for advanced breast cancer patients as a way of reciprocity.
Lack of awareness and limited mobility options for caregivers	▪ The social support that has to be provided by the family and acquaintances is marked as the major factor influencing the experiences of caregiving while dealing with patients of advanced breast cancer.
Financial responsibility and strain experienced by caregivers	▪ Relatives showed their concern over expense of treatment and inability to bear the expenses
Myths of beauty and shyness influencing caregiving attitudes and decisions	▪ Breast-related issues were not publicly shared with parents, daughters, or other relatives. ▪ People do not like to discuss such things publicly. ▪ Shyness and concept of veil hinders the communication of disease
Stressful and emotional experience of caregivers	▪ Reluctance to tell the real state of the disease to the patients ▪ The acceptance of disease by the patients and even for family member is a big dilemma ▪ Respondents directly and indirectly highlighted that males of the family have different and exacerbated pressure while giving care to women in their families with advanced breast cancer. ▪ Broken marital bond due to the burden of caregiving has also been highlighted while taking about how men deal with caregiving to advanced breast cancer patients.
Impact of treatment perspectives shaped by surrounding social groups	▪ Greater motivation to get treated if family members also have cancer ▪ Ignoring a clot in the breast region because no pain did not worsen. ▪ Knowledge about treatment options results in higher accessibility to treating patients
Challenges in consulting with male physicians and the associated stigma	▪ High demand for female healthcare workers ▪ Patient and caregivers feel more comfortable and secure with female physicians ▪ Stigmatization of breast cancer
Role of religious beliefs in caring for advanced breast cancer patients and in managing stress	▪ Closeness to God because of mental health stresses ▪ Coping mechanism for depression through religious prayer ▪ Respondents returned with responses of coping strategies. The responses mainly focus on religiousness to cope with the burden of care taking.

### Social responsibility and cultural reciprocity norms

This theme centers on the perceived social obligation of caregiving. Our findings indicate that in the local culture, caregiving responsibilities often fall on mothers and elder female relatives. Female respondents particularly noted it as their traditional duty to care for sick family members. Moreover, cultural acceptance of this responsibility was evident, especially in the case of married female caregivers (daughters and sisters), who were allowed by their in-laws to care for patients, a practice not typically facilitated under normal circumstances. This exception to the norm underscores the recognized duty of caregiving within familial relationships. Illustrating this point, a participant in the study shared:

My in-laws are very authoritative, but they allowed me to move to my parents’ house to take care of my mother as an elder daughter.

This statement reflects the cultural complexities surrounding family caregiving responsibilities, particularly for married women, and highlights the adjustments families make to honor these responsibilities.

Another major dimension was the primary motivation for some respondents to take on the caregiving task as a norm of reciprocity. The majority of respondents (12 out of 15) indicated that caring for the patient was a way of repaying a good deed they would receive from the patient or afterwards by God:

I bring her here, as it is my responsibility to take care of her as an elder son. Though it is a truth that at the moment I don’t have any other option, but I believe her prayers and blessings from the people around are big reward that will pay back to me in some form.

Thus, caregivers often feel a deep sense of obligation due to existing familial experiences and the established norms of caregiving in their culture. This responsibility is underscored by a general belief within the family that caregivers will eventually receive rewards for their kindness, viewed in a spiritual or moral context.

### Lack of awareness and limited mobility options for caregivers

In Pakistan, a low-income country, several social, economic, and cultural factors complicate caregiving. Primary among these is the distance to healthcare facilities, inadequate transportation, and limited resources for travel. One respondent shared:

We had to travel a long distance to get treatment for my mother. We live in a small town and we have little or no healthcare facilities there.

This not only imposes financial burdens due to multiple visits but also creates a stressful environment due to the lack of disease awareness, leading to confusion in emergency situations. One caregiver shared:

I have no idea about breast cancer, just heard about it in the family. The fear and misinformation surrounding the disease devastate my decision-making ability in emergencies.

For that reason, the decision for proper treatment in general and for emergency treatment in particular is associated with the level of awareness of the caregiver and the people around. Moreover, the fearful attitude towards cancer by relatives also makes caregivers confuse and stressful that it became difficult for them to handle the care of the patients.

### Financial responsibility and strain experienced by caregivers

The financial burden of treatment, including emergency care, places a significant strain on caregivers. Furthermore, limited social and home care resources to sustain their caregiver position generated a challenge resulting in changes in marital bonds, job capacities, lifestyles and perceptions of relationships. In some instances, this strain resulted in marital breakdowns and familial alienation. A participant recounted:

Ever since we told her husband about her breast cancer, he has not returned from Saudi Arabia. He has not even tried to contact his wife after that. He couldn’t bear the financial burden of treatment.

The socioeconomic conditions of the region are also a triggering element leading to the exacerbated stress among couples and resulting in drifts between them which may lead to separation as well. Moreover, the existing cultural practice of sending females back to their parental house for the care endorsed the existing norm and promotes the avoidance behavior of taking the responsibility for a sick person. In such cases, it becomes an obligation for parents and specifically male elders to take the responsibility:

She is my sister. I can’t throw her out of home like her husband. I have a strong blood relation with her, so it is a compulsion on me to take care of herself. Although my physical and mental health has been adversely affected in this situation.

### Myths of beauty and shyness influencing caregiving attitudes and decisions

Many Pakistani women share the traits of shyness and personal dignity. Few respondents shared that issues related to women’s breasts were often not openly discussed due to shyness and cultural norms. Many women are hesitant to undergo examinations by male practitioners or discuss breast-related issues openly. with parents, daughters, or other relatives. Owing to a lack of knowledge and an appropriate venue for open dialogue on these matters, participants agreed that the Pakistani population may not know enough about breast health. Therefore, they were unable to report their problem properly to the family members which leads towards a delay in diagnosis and treatment. When asked about the existence period of cancer treatment in this context, one of the participants responded:

She [her mother] did not start her treatment for 8 years. She did not share her symptoms with anyone due to shyness. She might share her condition with my father after a long time when symptoms were visible (in the form of bleeding from the nipple) but he wanted to consult a female doctor. Just few months back, we have started the treatment in a proper hospital as her illness has exacerbated.

Another respondent further mentioned:

I looked for a female doctor, so I came to Gangaram Hospital for treatment as I heard in this hospital only females treat the patients.

The same study participant also shared her concern regarding the cultural norms of Pakistani society:

Women often feel shy to discuss this disease with anyone. Social pressure may be one of the reasons that restrict women to go for treatment. People relate this disease with feminism and beauty. They mention breast removal and hair loss as loss of femininity of women body. People show their concern and sympathetic behavior in such a weird manner that makes the patient and her family much uncomfortable.

The social dynamics of shyness, *purdah* (veil) in the region, and the developed standards of beauty attached with women acts as a possible obstruction in the communication regarding the disease [35]. Resultantly, the decision of mastectomy (removal of breast) even creates a great debate among caregivers and family members. It shows that the cultural norms surrounding femininity, beauty, and modesty significantly affect communication about the disease and decision-making regarding treatment.

### Stressful and emotional experience of caregivers

Many caregivers admitted to concealing the severity of the disease from the patient, adding to the emotional burden. Moreover, few respondents related it to their own mental state and condition being the caregivers that had been affected adversely after the diagnosis of the disease:

I thought to tell her [sister] about the severity of the disease, but when I look into my own depressing state after doctor told me that her tumor has been spread into bones, I could not make my mind to tell her truth. She is already in pain. How could I increase her pain by telling this?

In this regard, another participant said:

She has a just five-year-old son, telling her about the disease is just like to ask her to die even before the day comes naturally. I don’t have courage to tell her the truth. I just told that it is a normal wound that will be managed after treatment.

The respondents also claimed that in the culture one usually hides the disease from the patient. Therefore, even some relatives who understood the extent and severity of the disease kept it to themselves. Knowing the severity means to open a new struggling phase for caregivers. The acceptance of disease by the patients and even for family member is a big dilemma.

Male caregivers often face increased stress, especially in caring for female patients, due to social pressures and financial burdens. Social pressure of relatives to go for alternative treatment such as spiritual healing and consultation to the traditional healers (Hakims) as well as financial burden make the situation stressful for them. In this regard, one of the participants who was an eldest son said:

I have become a rolling stone in making the decision of treatment for my mother being the eldest son. As paternal side were saying ‘Don’t go for an operation’, from the maternal side they said to go for operating. Then two or three relatives asked to go for some Darood and we tried it, but the disease increases so we again go to the doctor. They said we have made the case complicated and now it needs surgery.

Participant’s response revealed that, as a non-spouse male caregiver of a woman with advanced breast cancer, he saw himself as his mother’s protector and agent. From the above response, it could also be seen that the non-spouse caregivers, particularly male caregivers, had more responsibilities to handle as compared to male spouse caregivers. Among others, these includes bearing the expense of treatment and the residences, taking care for other people of the family, and balance their professional life in the time of extreme pressure.

A participant who brought his mother to the health facility for a chemotherapy session shared how his father was supportive towards his mother’s illness. In this case, the spouse caregiver said he made decisions with mutual understanding of the patient. But such examples are rare in the cultural context within Pakistan. Usually, women are not taken into consideration in the decision-making process in general and specifically for medical treatment.

The common experiences of respondents showed that a persistent or fatal illness, such as advanced breast cancer as well as its related sufferings would put the pair’s marital promise of “in times of sickness and health” to testing. Respondents described the challenging role of providing care and the complications involved with managing advanced breast cancer complications and medication-related harmful impacts at home as a challenge to their marriage ties, prompting them to consider separation.

In contrast, female caregivers expressed stress from caregiving and societal pressures, often receiving unhelpful advice from relatives. One respondent shared:

Even relatives sometime gave strange advises that I thought without having any prior experience how they can suggest us to give the patient herbal medicine, keeping them in a separate dark room without light for month for the treatment.

The social pressure and the discouraging attitude of relatives make family caregivers stressed, which is adversely impacting on their decision-making power and mental health. The very next theme explained this experience of caregivers.

### Impact of treatment perspectives shaped by surrounding social groups

In cases of advanced breast cancer, accessing treatment options and determining the importance of hospital care can be differentiated on the basis of the caregiver’s social groups. When asked about this phenomenon, one caregiver showed on hearing about a lump in the breast of their family member:

We knew that a lump was present, but because there was no pain, we ignored it. The pain was on and off, but never worsened, so we did not think about going to the doctor until later.

Respondents also shared that while telling the issue to close relatives, it was said that these are common symptoms of the post menstrual cycle and menopause that will relieve with the passage of time. These arguments convinced many caregivers. It indicates that caregivers were ready to avoid known symptoms of breast cancer, as lack of social exposure results in a decreased perception of severity of disease and threat. Social group experiences determine the motivation to get treated in the patient as well as the caregiver:

We were very in control with the treatment process and I wanted her to get treated as early as possible. She told me at night that she could feel a lump in her breast, and in the morning, we went to the doctor. It is because we have a relative [uncle] who also had cancer, so we knew that it is very important to start treatment early.

In Pakistan, having greater knowledge on treatment options for cancer results in positive decision-making to start treatment early. Comparing this to the findings, it can be argued that if an individual in the surrounding social group has had cancer, then knowledge on treatment options considerably increases, associated risks are identified and, therefore, treatment is accessed as early as possible.

### Challenges in consulting with male physicians and the associated stigma

The presence of a female physician imparts a sense of safety among the female population in developing countries, including India and Pakistan. This sense of security from having a female physician is driven due to the underlying socio-cultural values and norms associated with female gender:

We always made sure that my mother was being handled by female doctors, because she felt more at ease and I felt that she was protected.

The sociocultural values for treatment of advanced breast cancer patients in Pakistan have a high demand for female healthcare staff members. As seen in the study, this demand is highly prevalent and still increasing. However, another participant also highlighted:

Normally, we see that women– especially the house wives– are not free to take any decision about their health and cancer treatment. Male members of our family decided when to approach the doctor and also whom should be consulted. As breast cancer is a highly personal matter, it was decided to consult female doctor only.

The narrated fact supports the argument that the stigmatization of the disease has led to social values enforcing women to not opt for treatments, accessing social programs and other activities for alleviation of the disease. Especially in areas with low education and low literacy rate, cancer patients were discouraged from treatment:

Some of the family members discouraged to go for treatment. They were of the opinion that the treatment will not be beneficial.

Therefore, the stigmatization of breast cancer leads to high demands for female healthcare professionals as well as family members and discourages patients from getting treatment.

### Role of religious beliefs in caring for advanced breast cancer patients and in managing stress

A major theme identified was the role of religion that facilitated caregivers in establishing their mental health:

It is obvious that you get depressed when someone so close gets advanced cancer, but I think I started praying five times a day and it got rid of my depressive thought.

This indicates that religion was used as a coping mechanism for stress under such conditions:

I was in prayer as much as I could be, because it brought me inner peace. I was able to accept what was happening to her and instead of being sad I got the motivation to move forward with the treatment.

Therefore, it can be argued that religion and religious belief plays a crucial role in establishing the caregiver’s experience in the local cultural context, as it alleviates sources of stress and ensures internal stability. Participants of the study further highlighted that this is why healthcare professionals had to become inclusive of spiritual healing alongside physical treatment regimes, as both mental, physical and psychological wellbeing of the patient can be achieved.

The reason for the religious beliefs’ importance has been given by another participant in the following manner:

I was mentally so unstable that I visited the doctor twice for my check-up. I was afraid that I might get this disease. This disease has such adverse effects on blood relations that they suffer mentally. It took more than two years to come back to normal routine. But this trauma brought me closer to Allah. I started to offer my prayers more regularly.

Considering this information, the toll on mental health along with external stresses of advanced breast cancer enforced caregivers to cope through religion. Therefore, the caregivers in their experience of handling a patient of breast cancer strongly believed on religious practices to cope with anxiety and stress. At various points of the cancer process, caregivers face several problems that can have a profound effect on their functioning and quality of life:

It is a trauma, a shock that is impossible to describe in words.

When asked about how they cope with the burden of caregiving, one participant responded:

I pray to Allah. This is what relieves me the most.

The social values that are inbuilt in the culture result in the development of an insecurity and traumatic aftereffect which is not communicated due to the socially developed barrier. The caregivers find solace and comfort using different strategies including those related to religion such as praying rather than sharing with someone or consulting some counselor for psychological wellbeing.

## Discussion

It is a well-established fact that health behaviors are profoundly influenced by economic, social and cultural contexts, especially in tradition-oriented societies where strong socio-cultural norms shape individual and familial behaviors [33]. This influence is particularly evident in family caregiving for breast cancer patients. Our research indicates that sociocultural factors significantly shape the experiences of family caregivers, not only leading to delayed patient treatment but also adversely affecting caregivers’ experience, motivation, and mental capacity to manage the situation [22].

Family caregivers in Pakistan, who are often in close contact with patients throughout their treatment, are typically the primary decision-makers regarding the treatment. Many studies found that sociocultural aspects play a crucial role in shaping the experiences of caregivers for advanced breast cancer patients [34]. The qualitative data revealed that the caregiving experience is complex and deeply embedded within the cultural belief system and social responses. Factors such as lower socioeconomic status, cultural norms of pardah (veil), shyness, stigmatization, and gendered ideologies significantly influence the experiences of both male and female caregivers.

In many cultures, caregiving responsibilities are gender-specific, with males typically assuming the role of decision-maker in the family [35, 36]. This cultural orientation places a significant burden on male members in terms of providing social and economic support [37]. In rural areas with low socioeconomic status, caregivers face considerable stress in managing both the patient’s disease and societal responses. Our findings indicate that caregivers often view their role as a form of generalized reciprocity, where they do not expect immediate returns but believe that their good deeds will be rewarded by God in the long run. Female caregivers often see caregiving as a social responsibility ingrained in the cultural belief that females should be cared for by other females. However, economic dependence usually falls on male family members [38].

As patients were dependent on these caregivers and economic head of the family so their inability to bear expense of treatment directly affected the decision of treatment of the patients and associated delay. In developing countries, poverty was also identified a major factor of delayed presentation by different social researchers [40, 41].

The study also found a lack of awareness about breast cancer among caregivers, leading to ambiguity about the disease, its treatment, and care practices [35]. Consequently, many caregivers resort to traditional spiritual healers, which can worsen the patient’s condition. Fatalistic religious beliefs also contribute to delays in seeking proper treatment, with caregivers often relying on meditation to alleviate stress [41].

Our data suggest that male and female caregivers face different responsibilities. In cases where husbands do not assume responsibility for treatment, elder brothers often take on the role of caregiver and bear the financial burden. The denial of responsibility is also associated with low income of husbands and cultural practices of sending wives back to their parents’ home in times of sickness further complicate the caregiving dynamics [42–44].

The sociocultural values also influence the decision of caregivers to consult only female physicians for the treatment. In Pakistani local context, females are often not permitted to visit male specialists and are limited by male family members. Then again, ladies themselves feel shy and less certain with regards to showing their private part for assessment to male specialists because of religion and other socially implanted standards and qualities [44]. A study in this regard conducted by Mansoor and Abid (2020) also reported the experience of Pakistani women in negotiating their beauty and femininity resulting from breasts removal in local context [45]. Other researches also associated it as a major problem in timely diagnosis and treatment of breast cancer patients in local context [46–48].

The associated shame, shyness and stigmatization are culturally driven factors that lead both male and female caregivers’ mindsets to consult female physicians only. In such cases, the search of female physicians may lead to a delay in the treatment. It was also found during the analysis that cultural myths of female beauty associated with female appearance was a factor that creates reluctance in the mind of patient as well as family caregivers in order to consult the physician [49]. In case of surgery and removal of breasts, it originates stress on the patient as well as on the caregiver who had to deal with his or her own stress and has to counsel the patient as well [50].

The strong religious belief is often used as a mechanism to purge the tension in different matters particularly in case of critical disease like cancer [51, 52]. Family caregivers admitted it as a mechanism to control their disease and doctors also state it as a tool to relieve, pain stress and anxiety. Religion and belief system had a significant role in individual’s decision making. Belief has the power to mould individual’s positivity or negativity towards certain phenomenon. Greater sense of spirituality may positively affect the quality of life and improve social support. However, certain folk beliefs and fundamentalist religious doctrines, sometimes, lead to worse outcomes. Especially, in case of cancer diagnosis and treatment when these intervene in individual’s decision to consult some doctor for examination. This type of belief was also found to be influential in delay in seeking medical treatment [53–55].

Religion plays a significant role in alleviating stress among caregivers. Spiritual healing and meditation are important parts of dealing with stress and critical situations in Pakistani culture. Therefore, it is advisable for government and policymakers to provide counseling services to patients and their attendants to accept the disease and adopt proper treatment modes, potentially lessening the reliance on sociocultural beliefs in chronic diseases.

### Strengths and limitations

The strength of this study lies in its inclusion of family caregivers of both genders from three different cities in Punjab province, offering a diversified perspective. The study population includes individuals from both rural and urban areas. However, like all qualitative studies, the results are not generalizable, particularly due to the specific socio-cultural context of the study. Nonetheless, the random selection of study participants is a major strength of our research.

A key strength of this study lies in its inclusion of family caregivers from both genders across three distinct cities in Punjab province, ensuring a diverse range of perspectives. Additionally, the study encompasses participants from both rural and urban backgrounds, enhancing its comprehensiveness. However, like all qualitative research, this study faces inherent limitations. The most significant of these is the non-generalizability of the results, attributable primarily to the study’s focus on a very specific socio-cultural context. While these findings provide in-depth insights into this particular context, they may not be applicable to different settings. On the other hand, the random selection of participants stands out as a major methodological strength, supporting the robustness and validity of our study’s findings.

## Conclusions

Family caregiving for breast cancer patients in Punjab is strongly influenced by sociocultural norms, belief systems, traditional gender ideologies, and the poor socioeconomic status of caregivers. The study concluded that caregivers face numerous challenges, including limited understanding, financial constraints, societal disapproval, delayed decision-making, and demanding circumstances. Some caregivers used religion as a stress management tool, while others sought help from spiritual practitioners. Family caregivers play a pivotal role in the timely treatment and care of patients with advanced breast cancer, highlighting the need for support and empowerment from patients, healthcare professionals, and legislators. Addressing issues such as poverty, low income, and raising awareness about the disease are crucial to ensuring timely medical consultation and treatment. The study emphasizes that understanding sociocultural beliefs and practices, as well as interpersonal relations of patients and caregivers, is essential in managing the disease effectively.

## Data Availability

Data is available from corresponding author upon reasonable request.
